# Salient beliefs related to secondary distribution of COVID-19 self-test kits within social networks

**DOI:** 10.3389/fpubh.2024.1337745

**Published:** 2024-02-27

**Authors:** Cedric H. Bien-Gund, Molly Sarbaugh, Lily Perrine, Karen Dugosh, Robert Gross, Jessica Fishman

**Affiliations:** ^1^Division of Infectious Diseases, University of Pennsylvania Perelman School of Medicine, Philadelphia, PA, United States; ^2^Public Health Management Corporation, Philadelphia, PA, United States; ^3^Annenberg School of Communication, University of Pennsylvania, Philadelphia, PA, United States; ^4^Department of Psychiatry, University of Pennsylvania Perelman School of Medicine, Philadelphia, PA, United States

**Keywords:** COVID-19, self-testing, social networks, secondary distribution, beliefs

## Abstract

**Background:**

Widespread access to testing is critical to public health efforts to control the COVID-19 pandemic. Secondary distribution of COVID-19 self-test kits, where an individual distributes test kits to others in their social networks, is a potential strategy to improve access to testing. In this qualitative study, we identified salient beliefs about distributing and accepting COVID-19 self-test kits within one’s social network, as well as ordering COVID-19 self-test kits from the government.

**Methods:**

We recruited 61 participants from a randomized controlled trial (NCT04797858) in Philadelphia, Pennsylvania to elicit beliefs about (1) distributing COVID-19 self-test kits within one’s social network, (2) receiving test kits from social contacts, and (3) ordering self-test kits from the government. Using validated, open-ended question stems, we identified the most common set of beliefs underlying attitudes, perceived norms (or social referents), and perceived behavioral control (or self-efficacy) toward each of these behaviors.

**Results:**

Twenty-seven out of 30 (90%) of participants who received self-test kits reported distributing the kits to social contacts. These participants described altruistic beliefs about giving others access to testing, and felt approval from family members, friends, and others in their social networks. When receiving test kits from social network contacts, participants described advantages of test kit convenience, but some voiced concern about test kit tampering and confusing instructions. Participants also described perceived logistic barriers to distributing and receiving self-test kits, such as delivering or transporting test kits, or finding time to meet. Participants who ordered test kits from the government also described increased convenience of test access, but described different logistic barriers such as delays in test kit delivery, or not receiving test kits at all.

**Conclusion:**

In comparison with government-ordered test kits, the secondary distribution of COVID-19 self-test kits raised unique concerns about test kit quality and instructions, as well as distinctive logistic barriers related to distributing self-test kits to network contacts, which were not raised for test kits ordered from the government. This study demonstrates that beliefs may vary depending on the type of testing behavior, and behavioral interventions may benefit from developing messages tailored to specific testing strategies.

## Introduction

Since the beginning of the COVID-19 pandemic, widespread access to COVID-19 testing has been a public health challenge. Self-testing for SARS-CoV2, the causative virus of COVID-19, whereby individuals collects, performs, and interprets a test by themselves, can facilitate increased testing in the community (1). Lateral flow assay SARS-CoV-2 antigen self-test kits (COVID-19 self-tests) have proliferated in popularity due to their inexpensive cost, convenience, and rapid return of test results. In 2022, the US government began offering free COVID-19 self-test kits through online mail-in order (2). However, access to COVID-19 self-tests has remained limited and may be particularly difficult among underserved populations with low access to health services (3).

One potential strategy to increase access to testing is the secondary distribution of tests, where an individual distributes multiple test kits to others in their social network, such as family, friends, colleagues, and household members. This distribution strategy may overcome logistic barriers to testing (e.g., knowing where to obtain testing), mistrust of health systems (through obtaining tests from a known, trusted source), and provide more convenient testing. This strategy has been effectively leveraged to increase HIV testing to reach underserved or otherwise hard-to-reach populations with limited prior testing (4–6). In an ongoing randomized controlled trial (C-STRAND trial: NCT04796758), we are presently examining this strategy with the distribution of COVID-19 self-test kits (7).

Guided by causal models that predict decision making and behavior (8–10), we conducted a qualitative study to identify common beliefs underlying the distribution of COVID-19 self-tests. These causal models, such as the Theory of Planned Behavior and the Integrative Model of Behavioral Prediction, hold that a person’s behavioral intention is the strongest predictor of that behavior, and the strength of intention to engage in a behavior is, in turn, explained by one’s attitudes toward that behavior, along with their subjective norms and perceived behavioral control (also called self-efficacy) for the behavior of interest (8–10). When applying these models, belief elicitation studies are utilized to identify the specific beliefs that underly each of these three determinants of behavioral intention (11). We followed validated approaches to conducting this belief elicitation study, which includes defining the behaviors of interest (12, 13).

We focused on three health behaviors associated with distribution of COVID-19 self-test kits. The first two behaviors focused on the dyadic relationship in secondary distribution of self-test kits: *distributing* self-test kits *to* social network contacts and *receiving* self-test kits *from* social network contacts. Thirdly, because self-test kits also became available through the US government during the study, we also elicited beliefs about ordering tests from the government.

## Methods

We randomly selected participants from one of three federally qualified health centers (FQHC) in Philadelphia, US (7). These FQHCs serve a racially diverse urban patient population and provide community-based primary care regardless of ability to pay or immigration status, and also provide specialty services for substance use disorders, chronic viral infections, and family planning. After enrollment, participants were randomized 1:1 to distribute either (1) self-test kits or (2) clinic-based test referrals to others in their social networks in the C-STRAND trial. Self-test kits consisted of commercially available rapid antigen tests used to detect SARS-CoV-2 under FDA emergency use authorization. The self-test kit was a standardized, commercially available rapid antigen test kit, manufactured by Ellume Health (Ellume United States, Frederick, MD). Clinic-based referrals consisted of referral cards to get tested at local health centers at no cost. The study received ethical approval from the Public Health Management Corporation and the University of Pennsylvania Institutional Review Boards. All participants provided written informed consent.

Eight weeks after randomization, participants were invited to respond to open-ended questions designed to elicit their beliefs (Supplementary Files 1, 2). Investigators aimed to recruit 30 participants from each arm of the trial based on sample size principles for qualitative research (14), which would allow us to elicit the most salient beliefs with reasonable confidence that our estimates were accurate within at least 8 percentage point (15). Trained study staff conducted individual telephone interviews with participants. The subset of participants randomized to receive self-test kits were asked questions about distributing self-test kits to others in their social networks. Participants from both study arms were asked questions about receiving self-test kits from a friend or family member and obtaining self-test kits from the government. To assess testing behaviors and beliefs after receipt of test kits or test referrals, we conducted interviews 8 to 12 weeks after participant randomization and enrollment.

We randomly selected 30 participants from the self-test arm, and 31 participants from the test referral arm. The 30 participants from the self-test arm completed belief elicitations specifically about distributing COVID-19 self-test kits. All 61 participants responded to belief elicitations about accepting self-test kits from social network contacts. All participants were asked if they had heard about ordering test kits from the government. We then asked additional belief elicitations about obtaining test kits from the government among those that reported this behavior.

With each participant, we conducted semi-structured individual interviews that applied a set of standardized, validated belief elicitation methods (16). Specifically, we used six open-ended question stems that are meant to adapted to the behavior of interest. There are two question stems validated to elicit the beliefs underlying each of the three determinants of intention: attitudes, perceived norms, and behavioral control regarding the behavior. To elicit the beliefs underlying attitudes, the stems are worded, “what would be good about…” and “What would be bad about…” (e.g., what was good about offering someone a home test kit? What was bad about it?). To elicit the beliefs underlying perceived norms, the question stems are worded to identify social referents by asking, “Who might approve of you …?” and “Who might disapprove of you…?” To elicit the beliefs that underlie perceived behavioral control, or self-efficacy, the question stems are worded, “What would make it difficult for you to…?” and “What would make it easier for you to …?” These questions examine perceived barriers and facilitators to the health behavior of interest.

We followed standardized analytic strategies for belief elicitation studies and analyzed the responses provided to each question separately (9, 12). We used thematic analysis of verbatim responses to identify and categorize beliefs. We computed the frequencies of each belief reported and categorized the frequencies in rank-order. The most frequently reported specific beliefs are considered to be salient beliefs for the population sampled. Beliefs that were reported only once were excluded from analysis.

## Results

We conducted individual interviews with a total of 61 participants, whose median age was 38 years, nearly two-thirds reported Black/African American race, and two-thirds were assigned female sex at birth (Table 1). Nearly half of all participants reported household annual incomes of less than $50,000 and household sizes of three or more people. Among the 30 participants who received self-test kits for distribution, 27 (90%) reported distributing them to at least one other person. The mean number of test kits distributed was 3.81 (Standard Deviation 1.39) test kits per person.

**Table 1:** Study participant characteristics (*N* = 61).

Characteristic	Total	Received self-test kits	Received clinic test referrals
	*N* = 61 (%)	*N* = 30 (%)	*N* = 31 (%)
Age (Interquartile range)	38 (IQR 27–51)	41 (IQR 28–52)	33 (IQR 25–49)
Gender identity			
Male	17 (28)	11 (37)	6 (19)
Female	43 (70)	19 (63)	24 (77)
Transgender	1 (2)	0 (0)	1 (3)
Race			
Black/African-American	38 (62)	19 (63)	19 (61)
Asian	2 (3)	0 (0)	2 (6)
White	15 (25)	8 (27)	7 (23)
Some other Race	3 (5)	1 (3)	2 (6)
Prefer not to answer	3 (5)	2 (7)	1 (3)
Ethnicity			
Not Hispanic/Latino	52 (85)	27 (90)	25 (81)
Hispanic/Latino	8 (13)	2 (7)	6 (19)
Prefer not to answer	1 (2)	1 (3)	0 (0)
Education			
Some high school or less	5 (8)	1 (3)	4 (13)
Graduated high school or equivalent	15 (25)	9 (30)	6 (19)
Some college	12 (20)	6 (20)	6 (19)
Graduated college	21 (34)	10 (33)	11 (35)
Advanced degree	8 (13)	4 (13)	4 (13)
Household annual income			
Less than $25,000	16 (26)	9 (30)	7 (23)
$25,000 - $49,999	14 (23)	5 (17)	9 (29)
$50,000 - $99,999	8 (13)	5 (17)	3 (10)
$100,000 and above	6 (10)	3 (10)	3 (10)
Prefer not to answer	12 (20)	7 (23)	5 (16)
Household size			
One person (Lives alone)	14 (23)	8 (27)	6 (19)
2 people	17 (28)	9 (30)	8 (26)
3–4 people	21 (34)	10 (33)	11 (35)
5 or more people	6 (10)	2 (7)	4 (13)
Employment status			
Unemployed	14 (23)	7 (23)	7 (23)
Employed	43 (70)	22 (73)	21 (68)
In school, not employed	1 (2)	0 (0)	1 (3)

Rows may not add to total N due to missing data.

### Beliefs about distributing self-test kits

Beliefs underlying attitudes: As shown in Table 2, participants (*n* = 30) who reported their beliefs about distributing COVID-19 self-test kits to other social network contacts were more likely to report perceived advantages than disadvantages. The most commonly reported advantage to distributing self-test kits mentioned was the feeling of altruism and benevolence from assisting others: that giving test kits “felt good to help someone” (43%) and that it “gives others access to testing” (30%). There were few perceived disadvantages to distributing self-test kits, and the most mentioned belief was a critique of the self-test kit itself: the self-test kit was described as having “confusing test instructions” (10%).

**Table 2:** Frequency of salient beliefs about distributing COVID-19 test kits to social network contacts.

	Frequency (*n* = 30)	Responses (%)
Behavioral Beliefs (Beliefs Underlying Attitudes)		
Advantages		
Feels good to help someone	13	43%
Give others access to testing	9	30%
Speed and ability to get tests sooner	3	10%
Knowledge of infection and limiting spread of COVID-19	3	10%
Disadvantages		
Confusing test instructions	3	10%
Needing to save test kits for myself	2	7%
Feeling of rejection	2	7%
Social Referents (Normative Beliefs)		
Approvers		
My family (parents, siblings)	11	37%
Everyone I know	10	33%
My friends	2	7%
My co-workers	2	7%
My spouse or partner	2	7%
Everyone in my household	2	7%
Disapprovers		
People who do not believe in COVID-19 or conspiracy theorists	5	16%
Family members	2	7%
Control Beliefs (Self-Efficacy)		
Facilitators		
Ease of use	4	13%
Others needing more test kits	3	10%
Others collecting test kits from me	2	7%
Barriers		
Delivering test kits to people	3	10%
Having a limited number of test kits	3	10%
Uncomfortable offering test kits	2	7%
Technologically complex	2	7%

Perceived norms about who would approve or disapprove: When asked who would approve of them distributing a test kit the most common response was family members (37%) and “everyone I know” (33%), followed by friends, co-workers, spouse/partners, and household members (7% each). There were few referenced disapprovers, and the most common disapprover were simply “people who do not believe in COVID-19” or “conspiracy theorists” (16%).

Perceived behavior control beliefs: When asked what they believed would make it difficult to distribute a kit and what could make it easier, there were relatively few perceived facilitators and barriers. The most commonly described facilitator was the “ease of use” of self-test kits (13%). Consistent with the advantages of helping others, 10% also indicated that “others needing more test kits” would facilitate giving test kits to others. Barriers included logistic concerns with distributing self-test kits, such as difficulty delivering test kits to people (10%) and concern that self-test kits would be too technologically complex for others to use them (7%). In addition, 10% indicated having a limited number of test kits would be a barrier to them distributing test kits, and 7% also indicated they would feel uncomfortable offering test kits.

### Receiving self-test kits from social network contacts

Beliefs underlying attitudes: Participants (*n* = 61) noted several advantages about receiving test kits were noted by participants (Table 3). The most common stated advantages included “having test kits at home,” and “knowing if I had COVID-19.” Respondents also indicated that receiving a test kit from someone they knew had additional advantages. Thirteen percent indicated they would “feel closer to the person who gave me the test,” and 7% that the “test is probably reliable if a friend gave it.”

**Table 3:** Frequency of salient beliefs about accepting home test kits from a social network contact.

	Frequency (*n* = 61)	Responses (%)
Behavioral Beliefs (Beliefs underlying attitudes)		
Advantages		
Good to have tests at home	26	43%
I would know if I had COVID-19	20	33%
Feel closer to the person who gave me the test	8	13%
Test is probably reliable if a friend gave it	4	7%
Disadvantages		
Test kits might be tampered with or opened	14	23%
The friend would not have as many test kits	11	18%
Not sure of test kit quality	3	5%
Social Referents (Normative Beliefs)		
Approvers		
Everyone I know	19	32%
My family	17	28%
My friends	10	17%
My spouse	7	12%
Disapprovers		
Friends and family who do not believe COVID-19 is important	2	3%
People who do not believe in masking or vaccines	2	3%
Control Beliefs (Self-Efficacy)		
Facilitators		
Home delivery	10	17%
I know where the test came from	9	15%
Barriers		
Concerns of tampering of test kit	12	20%
Feeling guilty if they need it instead	6	10%
Concerns of spreading COVID-19	5	8%
Difficulty obtaining the test from a friend/ not having a car	4	7%
Finding time to meet friend	4	7%

Perceived norms about who would approve or disapprove of receiving test kits: The most reported approvers were “everyone I know” (32%), family (28%), friends (17%), and spouses (12%). Respondents reported that the perceived disapprovers would be those who either did not believe that COVID-19 was important (3%) or that masks or vaccines worked (3%).

Perceived behavior control beliefs: When asked about what could make it easier to receive home test kits from social network contacts, 17% mentioned having test kits delivered to their home and 15% also commented that “knowing where the test came from” made it easier to accept test kits. Obtaining self-test kits from social contacts had disadvantages as well: 23% also described concerns that the self-test kit “might have been tampered with,” and 5% described being “uncertain of the quality.” Respondents also noted they would “feel guilty if they (a friend) needed it instead” (10%) and concerns regarding COVID-19 exposure when accepting the test kit (8%). Others reported logistic barriers such as finding time to meet a friend to accept the kit (7%) and having a car to obtain the test kit (7%).

### Ordering test kits from the government

A total of 50/61 (79%) individuals had heard about ordering home test kits from the government, and 32/61 (52%) had ordered test kits and were asked additional questions regarding their experience ordering test kits from the government.

Beliefs underlying attitudes: Participants noted multiple advantages related to test kits being convenient to order and free (Table 4). The most common advantages of ordering tests from the government were having an easy online interface (38%), the testing was free (22%), and it was reassuring to have test kits (22%). Reported disadvantages were related to logistic barriers, such as having to “wait for test kits after ordering” (19%), “test kits never arrived” (9%), “needing a computer” (6%), and the household limit on test kits (6%).

**Table 4:** Frequency of salient beliefs about ordering COVID-19 test kits from the government.

	Frequency (*n* = 32)	Responses (%)
Behavioral Beliefs (Beliefs Underlying Attitudes)		
Advantages		
Easy online order interface	12	38%
Free	7	22%
Reassuring to have test kits	7	22%
Do not need to leave house to obtain	6	19%
Fast delivery	3	9%
Disadvantages		
Waiting for test kits after ordering	6	19%
Never received test kits	3	9%
Needing a computer/device	2	6%
Household limit of test kits	2	6%
Social Referents (Normative Beliefs)		
Approval		
My family (parents, siblings)	12	38%
Everyone I know	11	34%
My friends	4	13%
My spouse or partner	3	9%
Disapproval		
People who do not believe in vaccines or that COVID-19 exists	3	9%
Control Beliefs (Self-Efficacy)		
Facilitators		
Easy to order	14	44%
Ability to re-order test kits	5	16%
Barriers		
Limit on number of test kits per household	6	19%
Test kits never got delivered	4	13%
Lack of communication about delivery	2	6%

Perceived norms about who would approve or disapprove of ordering test kits from the government: The respondents most commonly noted family members (38%) and everyone they knew (34%) as approvers. When asked who would disapprove of ordering test kits from the government, most participants indicated no one would disapprove, or they cited people who did not believe in vaccines or that COVID-19 is important (9%).

Perceived behavior control beliefs: When asked what could make it easier to order test kits from the government, participants noted the test kits were easy to order (44%) and that they could re-order the test kits. Barriers to government delivered test kits included the limit on the number of test kits allowed per household (19%) and several participants again mentioned that the test kits never got delivered (13%), and lack of communication (6%).

## Discussion

This study applies a theory-based approach to understand a health-promoting behavior, COVID-19 self-testing, in an historically underserved urban population. This study is novel because it examines beliefs about an innovative strategy for distributing tests that relies on a dyadic relationship between a “index” (person who *distributes* a self-test) and “social network contact” (person who *receives* a self-test). We also used the theoretically ground approach to understand their beliefs about obtaining self-test kits online from the government. By comparing the belief sets for each of these different testing behaviors, this study provides important insights into the beliefs that vary, depending on the type of testing behavior.

We found that participants who received test kits for distribution overwhelmingly described altruistic beliefs, with the most common advantages being that it felt good to help someone and that they could give others access to testing. Public health messaging that emphasizes altruism have been utilized to promote COVID-19 vaccine uptake (17–19), but to our knowledge, has yet to be applied to secondary distribution strategies. We also found that altruistic beliefs were reflected in the set of perceived control beliefs reported, with some respondents describing the perception that others needed more test kits. These beliefs may be especially pronounced when the other individuals in need are friends, family members, and others closely related in their social network, whom participants felt would be supportive and approve of their distribution. In addition, some individuals describing the advantage of increasing knowledge of infection and limiting the spread of COVID-19. Very few participants spoke of a need to save the test kits for themselves.

We also observed that, for all three health behaviors (distributing self-test kits, receiving self-test kits from social network contacts, and obtaining self-tests online from the government) participants believed that many people would approve of them performing these behaviors. In all three behaviors, participants indicated approval from the same set of social referents, such as family, friends, co-workers, and “everyone I know.” Disapproval was rarely reported and limited to people who did not believe COVID-19 or its control measures were important.

When receiving test kits from social network contacts, we found that participants voiced concerns about tampering, confusing test instructions, and test kit quality. In comparison, among the subset of participants who ordered test kits from the government, there were no concerns about test kit quality or tampering. There are several potential reasons to account for this difference. First, participants who ordered test kits from the government were already motivated to obtain self-test kits and likely felt more comfortable with self-testing, compared with individuals who may be more ambivalent or unsure about self-test kit quality or how to use them, if they received them from a social network contact. Second, participants who received test kits from social network contacts may feel less certain that test kits were not tampered with or opened compared with if they received them from the mail. These concerns may be unique to the secondary distribution strategy, where individuals do not obtain test kits from a usual source, such as a health care setting or a government-sponsored program, where there may be fewer concerns about quality assurance. At the same time, some participants felt reassured if the test kit from someone they knew and felt the test would be reliable if it came from a friend. These findings suggest that secondary distribution interventions may need to emphasize test characteristics such as test quality assurance.

Participants described logistic concerns with all three health related behaviors, although different barriers were noted for government-ordered test kits compared with secondary distribution. Among individuals who received test kits for distribution, delivering the test kits to others was the most common barrier to distributing them. Similarly, when receiving test kits, participants noted difficulty obtaining the test from a friend, not having a car to get them, or finding time to meet a friend. In comparison, among participants who ordered test kits from the government, the most common barriers were having to wait for the test kits and a few individuals described never receiving the test kits. These data suggest that unique logistical concerns may arise for different modes of self-test kit distribution.

Several major limitations are noted. First, these results should be contextualized in the setting of an ongoing COVID-19 clinical trial taking place in an urban environment, limiting its generalizability. Clinical trial participants may be more motivated to perform health behaviors compared with the general population. Furthermore, in addition to different behavioral beliefs, rural residents may encounter different logistic barriers to distributing and obtaining self-test kits compared with urban residents if residences are less clustered. Second, we acknowledge that beliefs related to COVID-19 control measures can change over time.

## Conclusion

This study characterizes salient beliefs related to distributing and accepting COVID-19 self-test kits to and from social network contacts, as well as obtaining self-test kits from the government, in an urban population. Belief elicitation studies are considered the first step toward developing theory-based, effective behavioral interventions (11, 20). Compared with self-tests distributed through government-sponsored programs, secondary distribution interventions may also need to address concerns regarding test quality assurance and the unique logistic challenges of distributing test kits within social networks. Additional quantitative research is needed to identify which beliefs predict the strongest behavioral intention, which can then be used to optimize behavioral messaging to support widespread self-test distribution.

## Data availability statement

CB-G and JF had full access to all the data in the study and take responsibility for the integrity of the data and the accuracy of the data analysis. The raw data supporting the conclusions of this article will be made available by the authors, without undue reservation.

## Ethics statement

The study involving humans was approved by Public Health Management Corporation. The study was conducted in accordance with the local legislation and institutional requirements. The participants provided their written informed consent to participate in this study.

## Author contributions

CB-G: Conceptualization, Formal analysis, Funding acquisition, Investigation, Writing – original draft, Writing – review & editing. MS: Formal analysis, Investigation, Writing – review & editing. LP: Formal analysis, Investigation, Writing – review & editing. KD: Formal analysis, Investigation, Methodology, Project administration, Supervision, Writing – review & editing. RG: Conceptualization, Formal analysis, Funding acquisition, Supervision, Writing – review & editing. JF: Conceptualization, Formal analysis, Methodology, Supervision, Writing – original draft.
