# Supplementary material for: Salient beliefs related to secondary distribution of COVID-19 self-test kits within social networks
Source: Front Public Health. 2024 Feb 27;12:1337745. doi: 10.3389/fpubh.2024.1337745 (PMC10927981; doi:10.3389/fpubh.2024.1337745)
Supplement: Supplementary file 1 [file Data_Sheet_1.DOCX]

**Supplemental File 1: Semi-Structured Interview script for C-STRAND: Self-Test Group**

1. How many times have you been tested at [name of clinic]? If you're not sure, you can just give me your best guess.
2. Where else have you gone to get tested for COVID-19?
3. Have you ever used a self-test kit (the kind that is available to use at home)?
4. [If they have used a self-test kit before] How many times have you ever tested yourself using a self-test kit?
5. How many other times have you been tested for COVID outside the home, like at a pharmacy or drive-thru or clinic, not including self- tests or tests at [name of clinic]?*
6. Have you had a job, or gone to a school, that has required you to get tested for COVID-19?
7. If you wanted to get tested today or tomorrow, what steps would you take? Walk me through what you would do.*
8. If you needed to get more home test kits, how would you get them?*

Test kits from the government:

1. Have you heard that you can order COVID-19 home test kits online from the government and have them mailed to you?
2. [If yes] Have you, or has anyone you live with, ordered home test kits from the government?
3. [If yes] What was difficult about ordering them?
   *[If no]* What would be difficult about ordering them?
4. What would make it easier to order them?
5. [If they have ordered self-test kits] What was good about ordering them?
   [If they haven't ordered self-test kits] What would be good about ordering them?
6. [If they have ordered self-test kits] What was bad about ordering them?
   [If they haven't ordered self-test kits] What would be bad about ordering them?
7. Who might disapprove of you ordering them?
8. Who might approve of you ordering them?

We want to understand how to improve testing for Philly. It would be great if we can understand your experience with testing and your thoughts about it.

A few months ago, at the [insert clinic name here] clinic, you were offered COVID self-testing kits so that you or others could test at home.

1. Do you remember how many of those test kits you took with you?
2. Some people kept all the test kits and some people gave boxes away to others.

How many test kits did you give away, if any?

1. [If they offered 1 or more] Did you offer to give someone a test kit who didn’t want it?
   1. [*if yes*] How many times did that happen?
   2. [*if no*] How many times did that happen, where you offered someone a test kit and they didn't want it?

It would be helpful to learn about any cases where you planned to give someone a test but the plans didn’t work out. Sometimes people forget or get too busy or lose a box.

1. What things made it difficult for you to actually give a test kit to someone?
2. It would be helpful to learn about any cases where you planned to give someone a test but the plans didn’t work out. Sometimes people forget or get too busy or loose a box. What things made it difficult for you to actually give a test to someone?
3. [*If they have ever offered to share a test*:] How did you feel the first time you offered someone a home test kit? *[If they only offered a kit only once, ask:]* How did you feel when you offered the home test kit?

Thinking again about the first time you offered someone a test kit:

- 1. What was bad about offering it ? [*If they only offered a kit only once, ask:]* What was bad about offering it?
  2. What was good about offering it?
  3. What made it difficult to offer the test to someone?
  4. What could make it easier for you to offer the test to someone?
  5. Who might think it was a bad idea for you to offer someone a home test kit?
  6. Who might think it is a good idea for you to offer someone a home test kit?

*If more than one test was given to someone else:*

1. Think about the tests you gave away. Who did you give a test kit? We don’t need to know their name; just their relation to you.
2. Why did you decide to give a test kit to each of those people?
3. If basically anything was possible, what would be the easiest way for you to get a home test kit?

Imagine the city had a van driving by your home today that has lots of small boxes with a free COVID test kit inside each box. These test kits allow people to test themselves at home for COVID and learn the results in 15 minutes.

The people in the van say you can take home up to *5 test kits*. They could give you one of these test kits, or a stack of them so that you would have many COVID tests to take home.

There are debates about whether it would be a good idea for the city to offer these free COVID tests for people to take home. It may not be a good idea, and we are trying to understand if that is the case. This means your honest opinion is very helpful and valuable. You could help Philly avoid a mistake or do a good thing.

1. If the city’s van is driving by your place today to give out these free test kits, is it likely or unlikely that you would take any?
2. [if no, skip] How many tests would you like to pick up?
3. [if they want more than one] What would you probably do with each test? For example, would you want to use them to test yourself, or someone else you live with?
4. [if they want more than one] Would you be likely or unlikely to give one of the tests to someone who doesn’t live with you?

Now imagine that you don’t have any of these home-testing kits, but a friend offers you a box so that you can test yourself for COVID.

1. How would you feel about a friend offering to give you a test kit?
2. Would you be unlikely or likely to take a test kit home with you?
3. What could be bad about accepting a test kit from a friend?
4. What could be good about accepting a test kit from a friend?
5. Who might disapprove of you accepting a test kit offered by a friend?
6. Who might approve of you accepting a test kit from a friend?
7. What could make it difficult to accept a test kit from a friend?
8. What could make it easier to accept a test kit from a friend?
