# Supplementary material for: Salient beliefs related to secondary distribution of COVID-19 self-test kits within social networks
Source: Front Public Health. 2024 Feb 27;12:1337745. doi: 10.3389/fpubh.2024.1337745 (PMC10927981; doi:10.3389/fpubh.2024.1337745)
Supplement: Supplementary file 2 [file Data_Sheet_2.DOCX]

**Supplemental File 2: Semi-Structured Interview script for C-STRAND: Test Referral Group**

1. How many times have you been tested at [name of clinic]? If you're not sure, you can just give me your best guess.
2. Where else have you gone to get tested for COVID-19?
3. Have you ever used a self-test kit (the kind that is available to use at home)?
4. [If they have used a self-test kit before] How many times have you ever tested yourself using a self-test kit?
5. How many other times have you been tested for COVID outside the home, like at a pharmacy or drive-thru or clinic, not including self- tests or tests at [name of clinic]?*
6. Have you had a job, or gone to a school, that has required you to get tested for COVID-19?
7. If you wanted to get tested today or tomorrow, what steps would you take? Walk me through what you would do.*
8. If you needed to get more home test kits, how would you get them?*

Test kits from the government:

1. Have you heard that you can order COVID-19 home test kits online from the government and have them mailed to you?
2. [If yes] Have you, or has anyone you live with, ordered home test kits from the government?
3. [If yes] What was difficult about ordering them?
   *[If no]* What would be difficult about ordering them?
4. What would make it easier to order them?
5. [If they have ordered self-test kits] What was good about ordering them?
   [If they haven't ordered self-test kits] What would be good about ordering them?
6. [If they have ordered self-test kits] What was bad about ordering them?
   [If they haven't ordered self-test kits] What would be bad about ordering them?
7. Who might disapprove of you ordering them?
8. Who might approve of you ordering them?

We want to understand how to improve testing for Philly. It would be great if we can understand your experience with testing and your thoughts about it.

Imagine that a public health clinic near you was offering free COVID self-testing kits so that you or others could test at home.

1. How many self-tests would you want to take home?
2. [if not zero] How many of the tests would you want to keep for yourself and how many would you expect to use on others you live with?
3. Are there other people who you don’t live with who you would likely give some of the tests?
   1. If so, how many tests would you likely give to others?

1. Think about someone who you would be most likely to offer one of these home tests. Who is this person? [we don’t need to know their name, just their relation to you, like a friend or co-worker.]
2. What would be potentially bad about offering to give him or her one of your free tests?
3. What would be potentially good about offering to give him or her one of your free tests?
4. Who would think it’s a bad idea to offer one of your tests to someone?
5. Who would think it’s a good idea to offer one of your tests to someone?
6. What would make it difficult to share one of your tests with someone?
7. What would make it easier to share one of your tests with someone?
8. How many tests would you probably want to give to people who you don’t live with?
9. Are there people in your life who probably wouldn’t want you to offer them one of these tests? Maybe they live with you or maybe they don’t.

*if yes*: About how many people in your life probably wouldn’t want you to offer them one of the tests?

1. If basically anything was possible, what would be the easiest way for you to get a home test kit?

Imagine the city had a van driving by your home today that has lots of small boxes with a free COVID test kit inside each box. These test kits allow people to test themselves at home for COVID and learn the results in 15 minutes.

The people in the van say you can take home up to *5 test kits*. They could give you one of these test kits, or a stack of them so that you would have many COVID tests to take home.

There are debates about whether it would be a good idea for the city to offer these free COVID tests for people to take home. It may not be a good idea, and we are trying to understand if that is the case. This means your honest opinion is very helpful and valuable. You could help Philly avoid a mistake or do a good thing.

1. If the city’s van is driving by your place today to give out these free test kits, is it likely or unlikely that you would take any?
2. [if no, skip] How many tests would you like to pick up?
3. [if they want more than one] What would you probably do with each test? For example, would you want to use them to test yourself, or someone else you live with?
4. [if they want more than one] Would you be likely or unlikely to give one of the tests to someone who doesn’t live with you?

Now imagine that you don’t have any of these home-testing kits, but a friend offers you one so that you can test yourself for COVID.

1. How would you feel about a friend offering to give you a test kit?
2. Would you be unlikely or likely to take a test kit home with you?
3. What could be bad about accepting a test kit from a friend?
4. What could be good about accepting a test kit from a friend?
5. Who might disapprove of you accepting a test kit offered by a friend?
6. Who might approve of you accepting a test kit from a friend?
7. What could make it difficult to accept a test kit from a friend?
8. What could make it easier to accept a test kit from a friend?
